# Safeguarding patient privacy in eHealth systems: Bridging theory and practice through a scoping review and healthcare survey

**DOI:** 10.1371/journal.pdig.0001325

**Published:** 2026-03-26

**Authors:** Michael Winter, Robin Kraft, Celine Belas, Manfred Reichert, Maximilian Ertl, Rüdiger Pryss

**Affiliations:** 1 Institute for Clinical Epidemiology and Biometry, University of Würzburg, Würzburg, Germany; 2 Institute of Medical Data Science, University Hospital of Würzburg, Würzburg, Germany; 3 Institute of Databases and Information Systems, Ulm University, Ulm, Germany; 4 Service Center Medical Informatics, University Hospital Würzburg, Würzburg, Germany; Shahid Beheshti University of Medical Sciences School of Dentistry, IRAN, ISLAMIC REPUBLIC OF

## Abstract

The integration of eHealth technologies in hospitals has transformed patient care while raising privacy concerns. To address the latter, this paper combines a scoping review with a survey of healthcare professionals to examine both theoretical and practical aspects of privacy in hospital eHealth systems. Publications from 2021 to June 2024 focusing on privacy in hospital eHealth systems were reviewed. Literature was retrieved from PubMed, IEEE Xplore, ACM Digital Library, and Web of Science, resulting in 1,556 initial records. Additionally, 122 healthcare professionals from Swiss and German hospital networks were surveyed using purposive and convenience sampling regarding their perceptions of privacy measures and implementation challenges. From 434 included studies, 339 focused on technical measures, 40 on organizational processes, 29 on patient perspectives and ethical considerations, and 26 on legal and regulatory aspects. Key technical advancements included blockchain and AI. The survey revealed that participants primarily associated privacy with confidentiality of patient data and protection against unauthorized access. Most identified insufficient training, lack of standardization, and inadequate existing measures as key implementation challenges, thus highlighting gaps between theoretical privacy concepts and practical implementation in healthcare settings. While technical solutions dominate the literature, the survey emphasizes the importance of staff perspectives, particularly regarding confidentiality, access controls, training, and standardization. An integrated framework addressing technical, organizational, and workflow-specific privacy measures is proposed to bridge the theory-practice gap. Future eHealth privacy frameworks should balance technological innovation with practical implementation considerations that incorporate healthcare professionals’ insights to effectively safeguard patient data.

## Author summary

In recent years, digital technologies have become an essential part of hospital care, offering new opportunities but also raising serious questions about how patient information is protected. In this study, we wanted to understand how hospitals actually handle privacy when using eHealth systems. We reviewed a total of 1,556 research records on privacy in hospital digital systems published between 2021 and June 2024, and also conducted a survey with 122 healthcare workers recruited through hospital networks in Germany and Switzerland using purposive and convenience sampling methods. Moreover, since participation was anonymous, voluntary, and no demographic data was captured, formal ethical approval was not required. We found that while many technical tools like blockchain and encryption are being developed, the real-world challenges healthcare staff face are often overlooked. Staff told us that unclear procedures, lack of training, and time pressure make it hard to fully protect patient privacy. Our findings show that improving privacy in healthcare is not just about better technology, it also requires better support for hospital staff, clearer policies, and stronger alignment with legal frameworks. We developed a practical framework with implementation examples for different resource settings to help translate privacy principles into actionable measures. We hope our work helps bridge the gap between theory and practice so that digital health systems can be both innovative and respectful of patient privacy.

## Introduction

The digitalization of healthcare has transformed traditional medical practices through the implementation of eHealth technologies [1]. These eHealth solutions enable healthcare facilities to deliver more efficient and personalized care through electronic health records (EHR), telemedicine, mHealth applications, and remote monitoring systems [2,3]. However, this digital transformation introduces additional challenges regarding patient privacy and data protection.

As health data becomes increasingly digitized and networked, hospitals face responsibilities to protect sensitive patient information from unauthorized access, misuse, and data breaches [4]. Effective data protection measures are essential not only for maintaining patient trust but also for complying with legal frameworks such as the General Data Protection Regulation (GDPR) [5]. These measures must also uphold principles of equity across diverse populations, considering socio-economic differences (e.g., culture, education, age, and gender) [6].

Despite the growing importance of privacy in eHealth systems, research gaps persist in this context [7]. Previous studies have focused on technological aspects such as encryption, blockchain, or AI-powered security solutions, while the perspectives of healthcare institutions have received less attention, including hospitals processing extensive sensitive patient data [8–10]. Furthermore, there exists a disconnect between legal frameworks and the practical challenges encountered by hospital staff in daily operations [5,11]. This separation of legal and technological considerations often overlooks implementation challenges and their impact on patient care.

Another critical gap lies in empirically investigating hospital staff’s perceptions and experiences with privacy measures [12]. The effectiveness of current data protection implementations and specific challenges faced by healthcare professionals remain insufficiently documented, making it difficult to develop evidence-based recommendations for practice [11,13].

For this reason, this paper addresses these research opportunities through a comprehensive approach combining a scoping review of literature with a survey of hospital staff, in which 122 participants took part. By integrating theoretical frameworks with practical experiences, the following analysis aims to develop a comprehensive understanding of privacy in eHealth systems and contribute valuable insights to advance this critical area of research in healthcare digitalization. The research was guided by two primary questions:

**RQ1** How is the protection of patient data privacy addressed in eHealth systems within the hospital environment?
**RQ2** What discrepancies exist between theoretical concepts and the practical implementation of privacy measures in digital health applications in the hospital context?

The scoping review presented in this paper synthesizes diverse sources to systematically categorize key privacy themes in eHealth services. The complementary survey assesses healthcare professional’s understanding of privacy measures, their perceived effectiveness, practical challenges, and improvement recommendations. Further, while recent syntheses (e.g., [7,8] provide extensive taxonomies of privacy-preserving technologies and AI-driven security mechanisms, they remain confined to conceptual and technical perspectives. The present study’s contribution lies in explicitly linking these theoretical and technological frameworks to the operational realities of hospital environments through an integrated methodology. By combining a scoping review with a survey, this work connects research trends on data protection and regulatory compliance with firsthand experiences from healthcare professionals, who are responsible for related implementation. This two-fold approach reveals gaps between theoretical privacy models and how they actually work in clinical practice, unraveling insights previous surveys have overlooked. In addition, this paper offers an evidence-based framework that bridges technological advances with real-world healthcare privacy implementation.

## Theoretical foundations in security, privacy, and data protection

In eHealth, security, privacy, and data protection are interrelated yet distinct concepts, which are essential for protecting patient information. Privacy represents an individual’s right to control how, when, and to what extent their personal information is shared. Connected to autonomy and individual rights, privacy ensures personal data is processed only with the data subject’s consent, forming a foundational ethical principle in data protection [14].

IT security ensures the confidentiality, integrity, and availability (CIA) of data through technical and organizational measures, including encryption, firewalls, and access controls. While privacy protects data subjects’ rights, IT security defends against threats such as cyberattacks and data breaches [14].

Data protection synthesizes these concepts through legal and technical measures regulating personal data handling. Frameworks like GDPR provide legal structures that safeguard privacy while ensuring appropriate IT security measures, protecting against misuse and unauthorized access [14,15].

**Privacy-by-Design and Implementation Tensions:** While IT security measures such as encryption can enhance privacy, comprehensive surveillance for security purposes can paradoxically compromise it. For example, activity logging may unintentionally disclose sensitive information, underscoring the need to balance security measures with privacy considerations [14,15]. Privacy-by-Design addresses this challenge by integrating data protection into technologies and processes from inception rather than retrospectively [16]. Privacy-by-Design encompasses fundamental principles including data minimization, purpose limitation, and Privacy-by-Default, ensuring users’ privacy is protected automatically [17]. This includes both technical measures such as encryption and access controls as well as organizational approaches fostering a data protection-oriented culture.

In digital health applications, balancing data protection with functionality has become increasingly critical. eHealth technologies such as mHealth applications offer significant opportunities for personalized diagnostics and therapeutics [18]. Privacy and data security are therefore not merely legal and ethical obligations but central to the acceptance and long-term success of these technologies.

**European Union: Rights-Based Approach:** The GDPR follows a comprehensive, rights-based approach, establishing data protection as a fundamental right. The GDPR applies across sectors and globally when processing data of EU citizens, establishing principles such as data minimization, transparency, purpose limitation, and accountability.

The regulation empowers individuals through rights including data portability, erasure, and access to personal data. With clearly defined requirements, the GDPR has established global standards for data protection [19].

**United States: Sector-Specific Approach:** The United States employs a sector-specific, minimalist approach with no uniform national data protection legislation. Instead, sectoral regulations such as the Health Insurance Portability and Accountability Act (HIPAA) govern health data. HIPAA applies to health plans, healthcare clearinghouses, and healthcare providers processing certain electronic transactions.

HIPAA protects Protected Health Information (PHI) by requiring covered entities to implement reasonable security measures. It restricts use and disclosure of health information without consent while strengthening individuals’ rights to inspect, obtain copies of, request transmission of, or correct errors in their health records [20].

**Comparing GDPR and HIPAA:** Key differences between these regulatory frameworks include:

- **Scope:** While HIPAA is limited to health-related data, GDPR covers all personal information regardless of context.
- **Individual rights:** GDPR grants extensive rights including the “right to be forgotten,” whereas HIPAA focuses on regulating health information handling by “covered entities” and permits disclosure under certain conditions without explicit consent.
- **Implementation approach:** GDPR integrates data protection measures into organizational structures and processes (Privacy-by-Design), while HIPAA primarily aims to protect data in established systems, adapting requirements based on practical experience and technological developments [21].

Despite these differences, both frameworks establish standards for secure handling of sensitive data, aim to increase public trust in information protection, provide mechanisms for sanctioning breaches, and emphasize the importance of organizational and technical data security measures [21].

**China and India - Emerging approaches:** China has adopted a hybrid approach combining EU and US elements while emphasizing state interests. The Personal Information Protection Law (PIPL) and Cyber Security Law incorporate GDPR-like elements such as consent, transparency, and data security principles. However, China prioritizes data sovereignty, requiring that important data remain within the country. While data protection for private companies is strictly regulated, state monitoring activities are largely exempt. China also relies on non-binding yet quasi-binding guidelines [22]. India lacked GDPR-comparable legislation until 2019. The Personal Data Protection Bill (PDPB) introduced in December 2019 established the Data Protection Authority of India (DPAI) and imposed stricter requirements for processing sensitive personal data including health information [23]. Key features include data localization requirements, explicit and informed consent, comprehensive sanctions, and Privacy-by-Design [24].

Implementation challenges include effectiveness of consent-based models in regions with low digital literacy, exemptions for state institutions, and difficulties adapting international models to local contexts [24].

**Emerging challenges:** The rapid development of Internet of Things (IoT) devices, wearables, and genetic testing technologies presents challenges to existing data protection frameworks. Many of these technologies fall outside established laws such as HIPAA, creating significant privacy risks. The GDPR addresses some of these gaps through its technology-neutral formulation, but the evolving technological landscape continues to present regulatory challenges.

In healthcare settings, these regulatory frameworks must be operationalized while balancing privacy protection with the needs of effective healthcare delivery and innovative technological implementation.

## Related Work

Research consistently demonstrates that privacy and security are central concerns in healthcare, particularly as digitalization accelerates [25]. In general, prior work can be grouped into four main dimensions: technical, organizational, legal/regulatory, and patient-centered [26]. Each dimension addresses different aspects of privacy in eHealth systems. Despite extensive research, these dimensions remain unevenly developed, with the literature dominated by technological approaches and limited integration of organizational or human factors.

**Technical Dimension:** Several studies focus on technological mechanisms to ensure privacy in digital health systems. [27] emphasize the need to distinguish between privacy, confidentiality, and security. The latter terms are often conflated in practice and highlight encryption, blockchain, and access control as essential components of secure data exchange. Big data analytics, another major technical focus, has been identified as an enabler and a risk factor for privacy. [28] show that while analytics can improve personalized medicine and cost efficiency, it simultaneously heightens exposure of sensitive health data, calling for integrated security frameworks. Similarly, blockchain-based architectures offer promising methods for secure record management, though large-scale deployment remains difficult. [29] identify scalability limitations in proposed blockchain systems despite advances such as off-chain storage and sharding. Parallel developments in AI introduce new privacy vulnerabilities even as they promise operational efficiency. [30] caution that large language models may compromise confidentiality through data leakage or biased output if privacy safeguards are not embedded by design.

**Organizational and workflow dimension:** While technical measures dominate the field, few studies systematically analyze how privacy protection is implemented in healthcare organizations. [31] highlight the persistent neglect of the human factor, finding that healthcare professionals often show limited awareness of privacy risks despite growing patient concerns. Their findings underscore that organizational policies, training, and resource allocation remain weak links between technical innovation and clinical practice. Similarly, [32] identified infrastructure limitations, technical difficulties, psychological obstacles, and workload pressures as key challenges hindering the full implementation of digital health technologies related to privacy.

**Patient-centered and Ethical Dimension:** Empirical studies on patient experiences reveal specific vulnerabilities. [33] show that inadequate access control in patient portals can compromise adolescent confidentiality, eroding trust in digital care systems. Ethical discussions similarly point to tension between usability, transparency, and patient autonomy. The transition to fully digital hospitals, as analyzed by [34], illustrates this trade-off: while comprehensive EHR infrastructures enhance accessibility, they also increase documentation burden and elevate privacy risks.

**Emerging and cross-cutting Technologies:** Mobile and AI-enabled monitoring tools further extend privacy challenges. [35] demonstrate that mHealth and sensor-based systems can improve predictive care yet often lack robust privacy management, creating new vectors for data misuse and reliability concerns. In this context, the author from [36] discusses how the introduction of AI raises novel privacy issues related to implementation and data security, whereas [37] highlights the need for balancing pragmatically the application of AI in healthcare privacy context.

Across these research strands, technical innovation remains the dominant focus, while organizational implementation, staff behavior, and the translation of legal standards such as the GDPR into hospital workflows receive limited attention. Existing studies describe technological solutions (e.g., encryption, blockchain, AI-driven security) but rarely examine how these frameworks interact with staff responsibilities or institutional practices in clinical settings. Our study advances this field by integrating a scoping review with an empirical survey of hospital staff, linking technological and regulatory frameworks to the operational realities of privacy protection. This combined approach addresses these dimensions of data protection, particularly the alignment between legal principles, technical design, and day-to-day implementation in hospitals, offering a more comprehensive understanding of privacy in healthcare.

## Materials and methods

### Scoping review design

The scoping review was conducted according to the Preferred Reporting Items for Systematic Reviews and Meta-Analyses Extension for Scoping Reviews (PRISMA-ScR; see S1 Checklist) guidelines [38]. A scoping review was chosen over a systematic review as it enables broader collection and categorization of literature in a heterogeneous research field where multiple perspectives (e.g., technological, organizational, legal, and ethical) need to be considered. This approach is particularly effective for identifying research gaps in the domain of privacy in eHealth systems within hospital environments.

Unlike systematic reviews, which aim to answer narrowly defined questions and assess the quality or effect size of comparable studies, a scoping review is more suitable when the evidence base is conceptually diverse, methodologically heterogeneous, and unevenly developed, as it the case for eHealth privacy research. The literature in this domain spans technical prototypes, policy analyses, and qualitative assessments, which do not lend themselves to standardized quality appraisal or quantitative synthesis. Therefore, the scoping review methodology was scientifically appropriate for mapping the full range of evidence, summarizing the types and sources of available information, and identifying gaps for future systematic investigation.

Note that this scoping review was not prospectively registered, as it originated from an internal exploratory mapping of the literature to inform subsequent research priorities. Following completion of the review, the authors decided to disseminate the findings through a peer-reviewed publication due to their potential relevance for the broader research community. No methodological deviations from the initial exploratory plan occurred beyond refinement of search terms and categorization criteria during the screening phase to ensure conceptual consistency.

**Eligibility Criteria:** The healthcare environment was defined as encompassing all settings where healthcare is delivered, including hospitals, clinics, and related facilities involved in prevention, diagnosis, treatment, and rehabilitation, together with the administrative and data systems supporting these services [39,40]. Consistent with this, it includes all interactions and processes between patients and healthcare facilities, as well as data exchanges among stakeholders such as insurance providers, pharmacies, and public health authorities [41–43]. eHealth was defined as the use of information and communication technologies for health, encompassing digital technologies and platforms that enhance medical care and streamline administrative processes, such as EHRs, telemedicine, mHealth applications, and healthcare information systems facilitating data flows between stakeholders [44,45].

Furthermore, the following operational definitions of keywords were considered: Privacy refers to an individual’s control over access to personal health information. Confidentiality denotes the obligation of healthcare institutions and staff to protect patient information from unauthorized disclosure. Finally, security encompasses the technical and organizational measures that ensure data confidentiality, integrity, and availability. These operational definitions guided literature inclusion, data coding, and interpretation of survey responses.

Moreover, the timeframe for the scoping review was deliberately limited to studies published from 2021 onward. This decision reflects the rapid technological and regulatory developments in eHealth and patient privacy during recent years. The COVID-19 pandemic, the rise of AI in healthcare, and the evolving data protection frameworks (e.g., adaptations of the GDPR and national data governance acts) have substantially transformed technical and organizational approaches to privacy protection. Including older studies risked overrepresenting outdated frameworks, pre-pandemic infrastructures, and early-stage technologies that no longer align with current clinical realities. Therefore, the restriction to 2021 and later publications ensured that the analysis captured the most recent trends, technologies, and challenges relevant to contemporary hospital environments while maintaining methodological coherence and relevance to current practice.

The following inclusion criteria were applied:

1. Publications analyzing patient data privacy protection in eHealth systems in hospitals
2. Studies on mHealth applications in hospitals and their privacy impact
3. Works examining patient data handling by health insurers or public health authorities in the hospital context with a focus on privacy
4. Studies addressing personal data as defined by the General Data Protection Regulation
5. Publications from 2021 onward to ensure currency
6. English-language publications only
7. Studies using qualitative, quantitative, or mixed-method approaches

Exclusion criteria were:

1. Publications that primarily focused on other aspects of eHealth or hospital management and only marginally addressed patient data privacy
2. Studies that described technical details, implementation challenges, or general eHealth benefits without an explicit analytical link to privacy (e.g., purely engineering-focused works on encryption algorithms, database architectures, or system performance metrics without discussing their privacy implications)
3. Publications with inaccessible full texts
4. Studies focusing solely on IT security aspects (e.g., intrusion detection, malware defense, network resilience) without connecting these measures to privacy protection or data governance
5. Non-peer-reviewed publications (opinion articles, commentaries, or gray literature)
6. Systematic reviews or scoping reviews (excluded from the main analysis but used to identify additional primary studies and inform thematic classification)
7. Retracted publications

In general, publications that primarily addressed other eHealth or hospital management domains (e.g., workflow optimization, telemedicine adoption, IT infrastructure) and mentioned patient data privacy only briefly (e.g., a contextual remark or without substantive analysis) were excluded. Similarly, purely technical or security-oriented papers were excluded when they lacked an explicit conceptual or empirical connection to privacy, as the review aimed to synthesize studies that directly engaged with privacy principles, implementation, or perception.

Although systematic and scoping reviews were excluded from the formal synthesis to maintain focus on primary evidence, they were nevertheless consulted as secondary sources to refine the search strategy, identify additional relevant studies, and inform the development of thematic categories. This approach ensured comprehensive coverage of the literature while preventing redundancy between prior reviews and the present analysis. All stages of the scoping review (e.g., title/abstract screening, full-text screening, data charting) were conducted by a single expert reviewer. No second independent reviewer was involved, and therefore no inter-rater agreement metrics (e.g., Cohen’s K) were calculated. To mitigate ambiguity during screening and categorization, an initial calibration phase was performed in which a subset of records was iteratively reviewed to refine inclusion criteria. In cases of uncertainty, ChatGPT (GPT-4o) was used as a decision-support tool to assist in clarifying definitions and categorization options; however, all final inclusion and exclusion decisions were made by the expert reviewer. A list of excluded works can be found in S4 Data.

**Information sources:** The following four databases were selected in the scoping review:

- **PubMed:** Selected for its extensive collection of biomedical and health-related literature [46]
- **IEEE Xplore:** Chosen for its technical and engineering publications relevant to eHealth systems and privacy measures [47]
- **ACM Digital Library:** Selected for research in health informatics and data protection technologies [48]
- **Web of Science:** Chosen to provide a multidisciplinary perspective and enable analysis of citation networks and research trends [49]

This combination of databases ensured comprehensive coverage of both technological and medical aspects central to the topic while maintaining a balance between thematic depth and practical feasibility.

**Search strategy:** The search strategy was designed to enable precise and comprehensive identification of relevant publications. The search string was based on three core terms: “eHealth,” “privacy,” and “clinic,” supplemented with synonyms and related terms to broaden the search scope. Boolean operators (AND, OR), phrasing, and wildcards were used in developing the search strings, which were tailored and tested for each database. The literature search was completed on June, 2024 (i.e., cut off date was June 11, 2024).

The following search strings were used across the four information sources:

- **Web of Science:** ((ALL=(“privacy” OR “data protection” OR “confidentiality”)) AND ALL=(“Electronic Health” OR “eHealth” OR “Health information system” OR “Healthcare technologies” OR “Electronic Health records” OR “mobile Health”)) AND ALL=(“clinic” OR “hospital” OR “clinic information system”)
- **IEEE Xplore:** ((“All Metadata”:privacy) OR (“All Metadata”:"data protection”) OR (“All Metadata”:"confiden- tiality”)) AND ((“All Metadata”:"Electronic Health”) OR (“All Metadata”:eHealth) OR (“All Metadata”:"Health information system”) OR (“All Metadata”:"Healthcare technologies”) OR (“All Metadata”:"Electronic Health records”) OR (“All Metadata”:"mobile Health”)) AND ((“All Metadata”:clinic) OR (“All Metadata”: hospital) OR (“All Metadata”:"clinic information system”)
- **PubMed:** ((“privacy”) OR (“data protection”) OR (“confidentiality”)) AND ((“Electronic Health”) OR (“eHealth”) OR (“Health information system”) OR (“Healthcare technologies”) OR (“Electronic Health records”) OR (“mobile Health”)) AND ((“clinic”) OR (“hospital”) OR (“clinic information system”))
- **ACM Digital Library:** (“privacy” OR “data protection” OR “confidentiality”) AND (“Electronic Health” OR “eHealth” OR “Health information system” OR “Healthcare technologies” OR “Electronic Health records” OR “mobile Health”) AND (“clinic” OR “hospital” OR “clinic information system”)

In general, no controlled vocabularies (e.g., MeSH terms in PubMed, IEEE Thesaurus, or ACM Classification terms) were used in the search strategy. Only free-text keywords were applied to ensure the inclusion of recent and unindexed publications.

**Selection process:** In general, one expert reviewer analyzed identified records and the tool Covidence was used for performing the scoping review [50,51].

The selection followed a two-phase approach:

- **Title and Abstract Screening:** Publications were screened against three criteria: focus on patient privacy, relevance to the hospital environment, and focus on digital health applications. Publications meeting all three criteria advanced to full-text screening.
- **Full-Text Review:** The previously defined inclusion and exclusion criteria were applied to complete publications to ensure only relevant studies were included.

All decisions were documented to maintain transparency and enable comprehensive recording of the selection rationale.

**Data charting and items:** Data charting was conducted in accordance with PRISMA-ScR guidelines to ensure systematic and transparent synthesis of information. Data extraction and management were performed in Covidence, which facilitated the structured processing and documentation of the literature. The data extraction template, including all fields and guiding questions, is available in the supplementary material (see S2 Checklist).

Each publication was assigned to one of four predefined thematic categories that together formed the coding framework:

- **Technical Measures and Innovations:** Technical privacy solutions (e.g., encryption, access controls, blockchain, artificial intelligence, federated learning, IoT, mHealth, or cloud applications).
- **Organizational Measures and Processes:** Privacy-related training, data protection officers, internal policies, and incident response strategies.
- **Legal and Regulatory Aspects:** National and international data protection laws, compliance strategies, Privacy-by-Design/Default, consent management, and regulatory challenges.
- **Patient Perspective and Ethical Considerations:** Patient rights, consent procedures, trust and transparency mechanisms, ethical guidelines, and doctor–patient relationship impacts.

The initial data extraction form was piloted with 15 publications to verify clarity, coverage, and interpretability. Following testing, questions were refined to eliminate ambiguities and ensure consistent data capture. Coding was performed using an iterative approach: when new or ambiguous cases emerged, definitions and decision rules were refined, and previous codings were reviewed for consistency.

In cases where ambiguity persisted, an additional analysis step using ChatGPT (GPT-4o) was employed as an additional interpretive aid to clarify category boundaries and definitions. However, the final classification decisions were made exclusively by the expert reviewer.

**Synthesis methods:** The synthesis of results is based on systematically collected data and follows the guidelines for scoping reviews as outlined in [38]. The extracted data were organized and categorized using a predefined framework, which included technical measures, organizational processes, legal aspects, and patient perspectives. This structured approach allowed for a narrative and descriptive analysis aimed at identifying central themes, recurring patterns, and existing research gaps.

**Study quality and validity:** A formal quality assessment or risk of bias evaluation was not conducted in this scoping review. In accordance with the PRISMA-ScR framework [38], the principal objective of a scoping review is to provide a comprehensive mapping of the research field, identify emerging themes, and highlight knowledge gaps, rather than to appraise or weight the methodological rigor of individual studies. The intent of this review is therefore to capture an overview of current research on privacy in hospital eHealth systems without excluding studies based on methodological heterogeneity.

Nevertheless, to ensure a baseline level of reliability and validity, only peer-reviewed publications indexed in established scientific databases (i.e., PubMed, IEEE Xplore, ACM Digital Library, and Web of Science) were included. This restriction implies that all analyzed studies underwent editorial and peer evaluation processes, allowing a reasonable assumption of minimum scholarly quality.

Further, given the scoping nature of this review, detailed classification of the included studies (e.g., study design or methodological approach) was not undertaken. Scoping reviews aim to map the breadth and diversity of available evidence rather than analyze methodological distributions [38]. Future systematic reviews could address this aspect in greater depth.

### Survey design and implementation

To complement the scoping review and capture practical insights, a survey in Germany and Switzerland was conducted to examine the perceptions and experiences of healthcare staff regarding privacy in digital health applications. This mixed-methods approach aimed to identify potential discrepancies between theoretical findings and practical implementation. The selection of these two countries was justified by their shared German-speaking context, comparable healthcare systems, and strong emphasis on data protection under the GDPR (Germany) and Federal Act on Data Protection (FADP; Switzerland) framework, while still differing in national implementation and institutional practices. This combination allowed for comparative insights within a consistent legal and cultural context.

In general, a non-probability sampling strategy combining purposive and convenience elements was used. The survey targeted employees working in hospitals and hospital networks, irrespective of their professional role or area of activity. Invitations were distributed via bulk email to all employees of a Swiss hospital affiliated with one of the authors, as well as to convenience contacts from similar healthcare institutions in Germany, aiming to capture a broad spectrum of professional perspectives. Participation was voluntary and anonymous, with no compensation offered. The survey was executed between November 5 and November 27, 2024.

In total, 129 responses were received, of which seven were excluded because the respondents were not employed in a clinical environment, resulting in a final sample of 122 participants. Participants represented varied levels of professional experience across different healthcare institutions. No formal sample-size rationale or power analysis was applied, as the study pursued an exploratory objective focused on mapping staff perceptions rather than hypothesis testing. The open online design may have introduced self-selection bias, particularly toward digitally engaged individuals with heightened interest in data protection, which is considered when interpreting results.

The questionnaire was created and administered using Microsoft Forms and developed in German to ensure accessibility to the German-speaking target group. It consisted primarily of closed-ended questions, including single- and multiple-choice items as well as Likert-scale formats, allowing for standardized analysis of perceptions and evaluations. Further, questionnaire items were developed according to these operational distinctions (e.g., items on encryption = security; staff access control = confidentiality; patient consent = privacy). Open-ended responses were examined through a descriptive content analysis. All free-text answers were systematically reviewed and inductively grouped into thematic categories to identify recurring patterns and core messages, allowing qualitative insights to complement the quantitative findings. To ensure instrument clarity and reliability, a pretest was conducted with five hospital employees. The pretest evaluated question clarity, relevance, length, and technical usability. Based on participant feedback, linguistic adjustments and structural improvements were implemented. This validation process enhanced content clarity, response consistency, and internal validity of the instrument.

Item generation was informed by themes identified in the preceding scoping review and by established frameworks on data protection and Privacy-by-Design in healthcare (e.g., [52,53]). Domains included confidentiality, access control, organizational policies, and staff responsibilities. Items were adapted and operationalized for clinical relevance in the German-speaking hospital context.

The complete questionnaire, including all items and results, can be found as supplementary information (see S1 Data and S2 Data for comprehensive overview of questions and results for the survey). Key examples of survey items include questions such as: “To what extent do you consider yourself responsible for protecting patient data in your current professional role?” (5-point scale from 1 = not responsible to 5 = very responsible) addressing professional responsibility; “Which of the following measures are you aware of in your hospital?” (multiple selection of technical and organizational measures) assessing knowledge of protective mechanisms; and “How could your employer better support data protection?” (open field) capturing improvement suggestions.

The statistical analysis plan was defined a priori and limited to descriptive statistics, no inferential testing was planned.

In general, the questionnaire was defined in order to capture the following four key areas:

- Work environment and professional background
- Understanding and evaluation of privacy in healthcare
- Knowledge of protective measures and experiences with data breaches
- Suggestions for improving privacy protection

Ethical approval was not required for this study in accordance with institutional and national research guidelines governing research with human participants, as the survey involved no intervention and no collection of personal or demographic data. Participation in the survey was fully anonymous and voluntary, and responses could not be traced back to individual participants or their employers. Prior to accessing the survey, participants were provided with an information statement explaining the purpose of the study, the anonymous nature of data collection, the voluntary nature of participation, and the confidential handling of all responses. Informed consent was implied by participants’ decision to proceed with and complete the survey.

## Results

### Scoping review

In this section, we present the results of our scoping review addressing the first research question: How is the protection of patient data privacy addressed in eHealth systems within the hospital environment? The complete list of identified publications is available as supplementary information (see S3 Data for a comprehensive overview of all identified records in the literature review).

**Selection and characteristics of evidence:** Our systematic literature search resulted in 1,556 publications across four databases: ACM Digital Library (599), PubMed (404), IEEE (316), and Web of Science (237). After removing 158 duplicates, 1,398 publications underwent title and abstract screening. This process resulted in the exclusion of 756 publications, while full texts of 20 publications were inaccessible.

**Fig 1:**
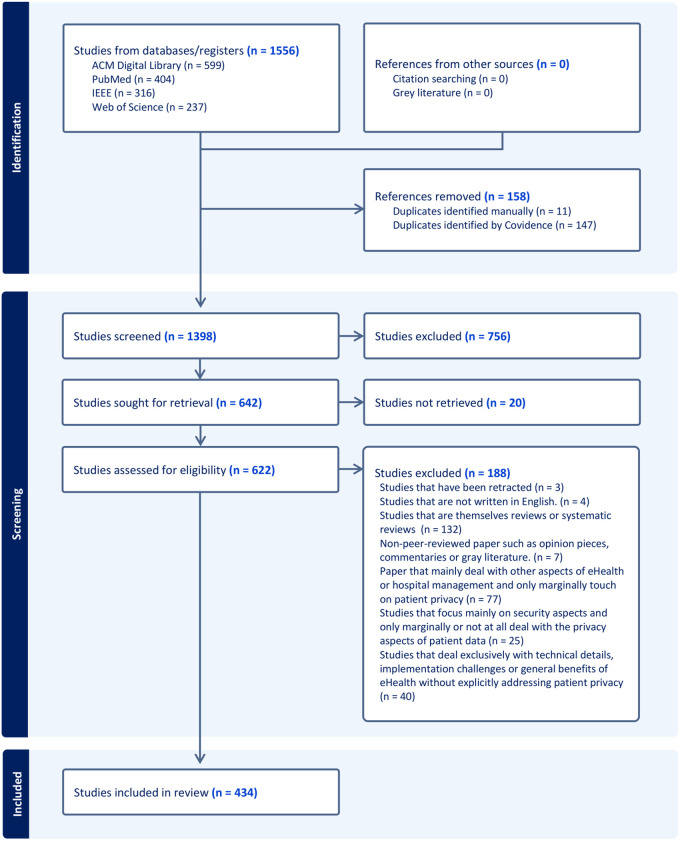
PRISMA-ScR Flow Diagram [38].

During full-text screening of the remaining 622 publications, we excluded 188 based on our predefined criteria: marginal reference to privacy (77), exclusive focus on technical details without privacy focus (40), focus on security rather than privacy (25), non-peer-reviewed literature (7), systematic or scoping reviews (32), retracted publications (3), and non-English publications (4). This rigorous selection process yielded 434 publications for final analysis. Fig 1 illustrates the review process as a PRISMA-ScR Flow Diagram.

The chronological distribution of publications demonstrated a consistent research interest in the topic, with 105 (24.2%) publications from 2021, 117 (27%) from 2022, 136 (31.3%) from 2023, and 76 (17.5%) from 2024 (until June 11, 2024). This represents an average of approximately 108 publications annually, suggesting sustained attention to privacy concerns in eHealth systems.

Geographical analysis revealed significant regional variations in research output. India led with 108 (24.9%) publications, followed by China (63, 14.5%)) and the USA (48 (11.1%)). At the continental level, Asia emerged as the most productive region with 185 publications. Within Europe, the United Kingdom (20 (4.6%)), Germany (14 (3.2%)), Italy (10 (2.3%)), and Norway (10 (2.3%)) were the primary contributors (see Table 1). This geographical distribution reflects varying regulatory frameworks, technological development priorities, and healthcare system structures across regions.

**Table 1:** Geographical Distribution of Included Publications (N = 434) by Top Contributing Country and Continent of Origin.

Continent	Country	Amount of Papers
Asia	China	63 (14.5%)
	India	108 (24.9%)
	Taiwan	14 (3.2%)
Australia	Australia	13 (3%))
Europe	Germany	14 (3.2%)
	Italy	10 (2.3%)
	Norway	10 (2.3%)
	UK	20 (4.6%)
North America	Canada	11 (2.5%)
	USA	48 (11.1%)
Others		133 (28.3%)

**Thematic analysis:** The 434 publications were categorized into four thematic groups: Technical Measures and Innovations (339 (78.1%)), Organizational Measures and Processes (40 (9.2%)), Patient Perspective and Ethical Considerations (29 (6.7%)), and Legal and Regulatory Aspects (26 (6.0%)).

**Technical measures and innovations:** Within the 339 publications categorized as Technical Measures and Innovations (multiple assignments of publications to specific domains were possible), blockchain technology emerged as the dominant approach with 166 publications. These studies frequently explored blockchain in combination with smart contracts to ensure secure data access and transactions. Encryption represented the second most prominent technical measure with 95 publications, while AI/ML approaches ranked third with 66 publications. Table 2 illustrates the distribution of these technological approaches across the analyzed literature.

**Table 2:** Number of Papers (N = 339) by key Technology Area Used for eHealth Privacy. Note that multiple assignments of publications to specific domains were possible.

Technology	Number of Papers
Blockchain	166
Encryption	95
AI/ML	66
IoT	60
Federated Learning	39
Smart Contracts	19
Cloud	13
HL7 FHIR	7
mHealth	6

The technical publications addressed several key challenges in eHealth privacy protection. A primary concern was safeguarding patient data from unauthorized access and misuse. [54] emphasized the importance of anonymization techniques and synthetic datasets to balance privacy protection with data availability for research and analytics. [55] highlighted how blockchain technologies could facilitate secure management of electronic health records, while [56] and [57] demonstrated the effectiveness of blockchain combined with attribute-based encryption for implementing fine-grained access controls.

Interoperability between heterogeneous systems emerged as another frequent concern in the technical literature. [58] illustrated how IoT-based blockchain solutions support privacy-compliant storage and management of electronic health records. [59] advocated for the adoption of standards like HL7 FHIR to facilitate secure and privacy to preserve data exchange across different healthcare information systems.

The generation of synthetic data and implementation of federated learning were identified as particularly promising approaches for enabling research while protecting patient privacy. [60] demonstrated how synthetic data generation could preserve statistical patterns while eliminating identification risks. Similarly, [61] and [62] showed how federated learning could enable collaborative model development without centralizing sensitive patient data, thereby maintaining both privacy and utility of the information.

These technical publications collectively demonstrate a strong research focus on innovative technological approaches to eHealth privacy. However, the predominance of theoretical and prototype-stage solutions raises questions about their practical implementation in clinical environments, particularly regarding scalability, cost-effectiveness, and integration with existing legacy systems.

**Organizational measures and processes:** The 40 publications focused on structural and procedural aspects of privacy protection in healthcare settings. These studies examined how operational frameworks, institutional policies, and human factors contribute to effective privacy safeguards beyond purely technical solutions.

Central themes in this category included the establishment of robust identification systems and access management procedures to safeguard sensitive patient data. [63] proposed comprehensive frameworks for managing data access based on role definitions and organizational hierarchies. [64] explored dynamic consent models designed to enhance transparency and patient control over information sharing, thereby strengthening organizational privacy practices.

The literature identified several barriers to effective organizational privacy measures. [65] highlighted the lack of standardization and interoperability between systems as challenges, particularly in multi-institutional settings. [66] documented the limited resources available to smaller hospitals for implementing comprehensive privacy programs. [67] examined cultural resistance to security protocols among healthcare staff, while [68] addressed infrastructural weaknesses in developing countries that impede effective privacy protection.

In response to these challenges, the literature proposed various interventions. [69] advocated for training programs designed to enhance digital literacy and privacy awareness among healthcare staff. [70] recommended the implementation of digital identity systems to manage access controls more effectively. [71] proposed dynamic threat detection systems that adapt to evolving privacy risks, while [65] emphasized the adoption of standards such as HL7 FHIR to improve interoperability while maintaining privacy protections.

Beyond specific interventions, these publications emphasized the importance of integrating privacy considerations throughout organizational structures and processes. [72] advocated for incorporating privacy requirements into the early development phases of eHealth systems rather than as after-the-fact additions. In turn, [64] proposed participatory design methodologies to ensure privacy measures reflect actual workflow needs. [73] explored the establishment of centralized data integration centers with standardized privacy protocols, while [69] emphasized the importance of fostering collaboration between IT departments and healthcare staff to develop practical privacy solutions.

These organizational-focused publications reveal a critical aspect of privacy protection often overshadowed by technological approaches in the literature, highlighting how institutional culture, workflows, and human factors significantly influence the effectiveness of privacy measures in healthcare settings.

**Patient perspective and ethical considerations:** Our analysis identified 29 publications in this context that are related to privacy in eHealth systems. These studies examined patients’ rights, needs, and expectations regarding the handling of their sensitive health data. Within this category (multiple categorization of papers), transparency and trust were the most frequently addressed topics (21), followed by confidentiality and protection of sensitive data (17), and patient rights and autonomy (11). Table 3 summarizes the distribution of publications across these patient-oriented and ethical focal points.

**Table 3:** Number of papers (N = 29) by Ethical and Patient-related Category. Note that multiple assignments of publications to specific domains were possible.

Category	Number of Papers
Transparency and trust	21
Confidentiality and protection of sensitive data	17
Patient rights and autonomy	11
Ethical guidelines and recommendations	5
Consent management	3
Discrimination and fairness	2
Long-term impact on the doctor-patient relationship	1
Conflicts between privacy and research	1

The literature documented patient concerns regarding potential data breaches and unauthorized access to their health information. [74] investigated patient perspectives on privacy risks associated with national patient portals and found that data security fears represented a barrier to adoption of these systems. Psychiatric data emerged as particularly vulnerable, with studies by [75] and [76] highlighting how security gaps could lead not only to identity theft but also to stigmatization and psychological distress for affected patients. Research by [77] and [78] found that patients frequently criticized the lack of transparency regarding how their data was processed, shared, and protected within healthcare systems.

Ethical dimensions of privacy protection received attention in the literature. [33] examined the unique privacy needs of adolescent patients, while [79] investigated privacy concerns specific to transgender individuals in healthcare settings. These studies underscored the importance of strengthening patient rights and autonomy, particularly for vulnerable populations whose privacy needs may differ from the general patient population. [80] demonstrated how co-design approaches that actively involve patients in developing privacy solutions could effectively enhance patient autonomy and address their specific concerns.

Trust and transparency emerged as central themes across this body of literature. [81] and [82] investigated the relationship between perceived privacy protections and patient trust in healthcare providers, finding that robust privacy measures enhanced patient willingness to share sensitive information. Studies by [83] and [84] highlighted concerns regarding the protection of sensitive data categories, including genetic information and mental health records, which patients perceived as requiring heightened privacy safeguards.

This patient-centered literature reveals important dimensions of privacy that extend beyond technical and organizational aspects. By examining privacy through the lens of patient experiences and ethical frameworks, these studies highlight the human impact of privacy measures and their role in maintaining trust, dignity, and patient autonomy within healthcare relationships.

**Legal and regulatory aspects:** Our analysis identified 26 publications focused on legal and regulatory frameworks governing health data protection. These studies examined how formal regulations shape privacy practices in healthcare environments and the challenges of achieving compliance while maintaining operational efficiency.

The GDPR emerged as the most frequently referenced legal framework, discussed in 15 of the analyzed studies. [85] and [86] explored variations in GDPR application across different European countries, highlighting challenges in achieving consistent implementation. In turn, [87] specifically examined GDPR compliance within NHS England, documenting both successes and ongoing difficulties in adapting healthcare processes to meet regulatory requirements.

In the United States context, the HIPAA dominated the regulatory landscape, appearing in 7 publications, while the 21st Century Cures Act was discussed in 2 studies. [88] analyzed challenges related to multi-user functionality of electronic health records and identified tensions between state regulations and federal frameworks. Further, [89] examined the integration of substance use disorder data into electronic health records, highlighting how the federated regulatory structure in the United States creates particular challenges for managing sensitive information while respecting privacy requirements.

As shown in Table 4, publications in this category focused primarily on Compliance/Implementation (26) and Legal Frameworks (26), followed by Privacy-by-Design (15), Consent Management (8), and International/Regional Differences (7). This distribution reflects the complex interplay between establishing regulatory standards and translating them into operational practices.

**Table 4:** Number of Papers (N = 26) by Legal and Regulatory Category. Note that multiple assignments of publications to specific domains were possible.

Category	Number of Papers
Legal framework	26
Compliance and implementation	26
Privacy-by-Design	15
Consent management	8
International and regional differences	7

International and regional differences in implementing data protection laws emerged as challenges in multiple studies. [70] and [90] documented how divergent regulatory standards complicated health data interoperability across borders, creating barriers to multinational research and care coordination. At the national level, [91] examined efforts to harmonize consent processes for health data sharing within Germany, demonstrating how regulatory requirements shape technological implementations.

The integration of Privacy-by-Design principles into technological systems received increasing attention in the literature. [85] highlighted the relevance of these principles for automated monitoring systems in healthcare, while [92] examined their application in integrating sensitive health data into electronic health records. These studies emphasized the importance of embedding privacy considerations from the inception phase rather than addressing them retrospectively.

The legal and regulatory literature reveals the critical role of formal frameworks in establishing standards for privacy protection while highlighting the complexity of implementing these requirements in diverse healthcare contexts. These publications provide essential insights into the regulatory foundations that shape both technological and organizational approaches to privacy in eHealth systems.

## Survey results

To address the second research question (RQ2: What discrepancies exist between theoretical concepts and practical implementation of privacy measures?), we conducted a survey with 129 healthcare professionals. After excluding 7 participants who did not work in healthcare settings, 122 (100%) valid responses were analyzed. Most participants (107 (87.7%) worked in Switzerland, while 15 (12.3%) were based in Germany. The sample included experienced healthcare professionals, with 69 reporting more than 11 years of experience in healthcare environments (see S1 Data and S2 Data for comprehensive overview of questions and results for the survey).

**Understanding and perception of privacy:** When asked about their understanding of privacy in digital health applications (multiple responses permitted), participants most frequently associated privacy with confidentiality of patient data (120 answers (98.4%)) and protection against unauthorized access (115 (94.3%)). Other common associations included ensuring data integrity (72 (59%)) and anonymization of health data (62 (50.8%)). These responses indicate that healthcare professionals conceptualize privacy primarily in terms of data security and confidentiality.

**Perceived importance of privacy in healthcare:** Participants prioritized multiple aspects, with protection of sensitive personal data (117 (95.9%)) and trust between patients and healthcare staff (101 (82.8%)) receiving the most attention. Compliance with legal regulations (95 (77.9%)), preservation of human dignity (72 (59%)), and prevention of security risks (72 (59%)) were also identified as important. These findings suggest that healthcare professionals have a multifaceted understanding of privacy that extends beyond technical security measures to encompass relational and ethical dimensions.

**Privacy breach experiences and responses:** Among the participants, 42 (34.4%) reported having experienced privacy breaches in their workplace. When asked about organizational responses to these incidents, participants most commonly reported contacting supervisors (25 (20.5%)) and implementing professional training initiatives (23 (18.9%)). Other answers included notification of data protection officers (16 (13.1%)), internal investigations (11 (9%)), and implementation of technical adjustments (8 (6.6%)). Notably, 9 (7.4%) participants reported being unaware of how privacy breaches were managed within their organization, suggesting potential gaps in incident communication protocols and highlighting the need for more transparent incident management processes.

**Privacy measures and implementation challenges:** Participants identified various privacy measures implemented in their institutions. Regular data protection training was most frequently mentioned (90 (73.8%)), followed by monitoring and logging of system access (81 (66.4%)), encryption of patient data (73 (59.8%)), role-based access controls (68 (55.7%)), and two-factor authentication (43 (35.2%)). This distribution suggests that healthcare institutions employ a combination of technological, procedural, and educational measures to protect patient privacy, with training and monitoring receiving particular emphasis.

**Ensuring privacy in their daily work:** Time pressure and excessive workload emerged as the most significant barrier (93 (76.2%)), indicating that privacy measures often compete with clinical demands for staff attention. Other obstacles included unclear guidelines and processes (42 (34.4%)), technical limitations of systems (42 (34.4%)), communication issues between departments (30 (24.6%)), insufficient training (27 (22.1%)), and limited management support (22 (18%)). The prominence of time pressure as a barrier suggests that privacy measures must be efficiently integrated into workflows to be effective in healthcare environments where competing priorities exist.

**Professionals attitudes and knowledge:** Participants rated the importance of privacy in healthcare very highly (mean = 4.76 (standard deviation (SD) = 0.56)), demonstrating strong recognition of its value. However, notably lower scores were reported for awareness of privacy issues among colleagues (mean = 3.50 (SD = 0.84)), personal knowledge about privacy measures and guidelines (mean = 3.52 (SD = 0.97)), and prioritization of privacy relative to other tasks (mean = 3.63 (0.85)). Interestingly, the effectiveness of current measures to protect patient data was evaluated as above-average between the participants (mean = 3.57 (SD = 0.75)).

When considering open-ended questions addressing how privacy can be improved for patients, several core themes emerge. Participants emphasize the need for continuous, practical, and organization-wide training to strengthen awareness and responsible behavior beyond IT departments. Access management is seen as critical, including the elimination of shared accounts, stricter access controls, and the implementation of two-factor authentication. Structural measures such as establishing or expanding information and data security management systems with clear roles, regular audits, and defined accountability are also highlighted.

Technical improvements (e.g., comprehensive data encryption, secured networks, avoidance of external data carriers) are viewed as essential, alongside physical and procedural safeguards like locked disposal containers, screen-lock policies, and facility adjustments to ensure privacy in open or crowded treatment areas. Respondents call for greater resource allocation and stronger engagement from leadership, including appointing data protection officers with real authority.

A transparent and consistent communication culture, where privacy is treated as a shared organizational value, is considered vital. Simplifying processes, digitalizing workflows, and integrating privacy into patient care and digital strategies are proposed to make compliance more practical. Finally, reducing workload and time pressure is seen as a necessary precondition for staff to apply privacy standards consistently and attentively in everyday clinical work.

These disparities highlight a gap between the acknowledged importance of privacy and its practical implementation in healthcare environments. While healthcare professionals recognize privacy’s significance in principle, they report only moderate levels of awareness, knowledge, and prioritization in practice. The thematic analysis suggests that this gap stems from competing operational pressures, inconsistent management commitment, insufficient resources, and limited or ineffective training structures. Together, these factors hinder the translation of privacy values into consistent, actionable behavior within the realities of clinical practice.

## Discussion

The integration of findings from our scoping review and healthcare survey provides comprehensive insights into privacy protection in eHealth systems within hospital environments. This dual-methodology approach reveals significant patterns, gaps, and implementation challenges that merit detailed examination. Moreover, previous surveys have primarily emphasized technological or algorithmic aspects of privacy in eHealth systems, focusing on encryption methods, blockchain frameworks, or AI-based security mechanisms without addressing their translation into hospital practice [7,8]. By combining a scoping review with a practitioner survey, this study extends beyond these technology-centered syntheses to connect theoretical concepts with operational experience. This integration reveals where privacy protection succeeds or fails in real hospital workflows, training practices, and governance structures. Accordingly, the following insights contribute a multidimensional understanding of privacy that unites regulatory, technical, and human factors, offering an evidence-based bridge between abstract data protection models and their practical realization in clinical environments.

**Research focus vs. Practical Needs - A Critical Misalignment:** Our analysis revealed a striking imbalance in research focus, with Technical Measures and Innovations dominating the literature (339 (78.1%) out of 434 publications), while Organizational Measures (40 (9.2%)), Patient Perspectives (29 (6.7%)), and Legal Aspects (26 (6%)) received comparatively limited attention. This distribution suggests a fundamental misalignment between research priorities and practical implementation needs in healthcare settings.

The predominance of technological solutions in the literature contrasts with survey findings, where healthcare professionals emphasized organizational, regulatory, and workflow-related challenges as primary concerns [93]. As a result, while researchers focus on developing sophisticated technical solutions like blockchain and advanced encryption, practitioners struggle with basic implementation challenges including insufficient training, unclear guidelines, and workflow integration difficulties.

**Technological solutions - promise and implementation barriers:** Technological approaches offer significant potential for enhancing data protection. Blockchain technology emerged as particularly promising in the literature, with 166 publications highlighting its capabilities for ensuring transparency, traceability, and secure transactions in health data management [27]. Similarly, AI-driven solutions demonstrated potential for optimizing access controls and identifying anomalous patterns indicating potential breaches [94].

However, our survey revealed considerable skepticism among healthcare professionals regarding the practical implementation of these technologies. Key barriers identified included:

- **Resource limitations:** Particularly in smaller facilities lacking specialized IT personnel and financial resources
- **Scalability challenges:** Several blockchain implementations demonstrated in research settings faced difficulties scaling to enterprise-level healthcare environments [29]
- **Integration complexities:** Implementing new technologies within existing legacy systems created interoperability issues
- **Technical maturity:** Many proposed solutions remained at theoretical or prototype stages, lacking robust real-world validation

These findings suggest that technological solutions, while valuable, require careful consideration of implementation contexts to be effective in practice.

**Organizational dimensions - the underestimated foundation:** Our review and survey highlighted the critical importance of organizational measures in establishing effective privacy protection. Survey participants emphasized that clear policies, regular training programs, and standardized privacy protocols formed the foundation of successful privacy protection—factors that received disproportionately little attention in the literature.

Healthcare professionals consistently reported challenges in integrating privacy measures into clinical workflows. Time pressure and workload emerged as the most significant obstacles, alongside with unclear guidelines and technical limitations. These findings align with previous research by [31] highlighting limited privacy awareness among healthcare staff due to insufficient organizational support.

**Regulatory compliance - navigating complex requirements:** Legal frameworks provide essential guidance for privacy protection, yet our findings indicate significant challenges in translating regulatory requirements into practical implementation. Survey participants reported difficulties interpreting complex regulations and determining appropriate technical and organizational measures to achieve compliance.

The geographical distribution of publications raises important questions about the transferability of findings across different regulatory contexts. Many technological solutions proposed in the literature, particularly those originating from India (108) and China (63) were developed under regulatory environments that differ from the GDPR and may not prioritize its stringent requirements. Consequently, these approaches, while technically innovative, may encounter limitations when applied in GDPR-governed healthcare settings, contributing to the observed implementation gap in our survey.

Publications from regions with comprehensive data protection regulations (EU, US) demonstrated greater emphasis on integrating legal requirements into technological and organizational measures and therefore provide more directly applicable insights for European healthcare institutions seeking to balance innovation with compliance.

**Socioeconomic and cultural influences on legal interpretation and acceptance:** Legal frameworks do not operate in isolation; their effectiveness depends on the social, economic, and cultural environments in which they are applied. Economic inequalities (e.g., differences in income levels, broadband access, availability of digital devices) directly affect individuals’ ability to use eHealth services and understand the legal conditions governing data protection [95]. Limited connectivity or outdated devices restrict participation in digital health ecosystems, leading to unequal awareness and comprehension of privacy rights [96].

Even in developed contexts like Switzerland and Germany, urban–rural disparities persist. Rural populations often face slower internet speeds, fewer digital health infrastructures, and limited access to training or institutional support. These economic asymmetries contribute to unequal enforcement of eHealth-related laws and varying capacities to exercise data rights effectively [97].

Educational and cultural systems further shape digital literacy and health literacy [98]. Populations with limited exposure to information technologies or weak formal education may struggle to interpret legal documents, comprehend consent mechanisms, or navigate privacy options embedded in eHealth platforms [99]. Consequently, digital and legal literacy gaps influence not only compliance but also the perceived legitimacy of privacy regulations.

Cultural differences in trust also play a critical role. In societies where confidence in governmental institutions or technology companies is low, individuals may resist data-sharing initiatives or perceive eHealth regulations as intrusive rather than protective [100]. Conversely, cultures with high institutional trust are more likely to accept centralized data management and regulatory oversight. These variations demonstrate that acceptance and enforcement of eHealth laws depend as much on sociocultural dynamics as on formal legal design.

**Ethical considerations and patient perspectives:** Both the literature review and survey responses highlighted tensions between privacy protection and competing values in healthcare. Healthcare professionals expressed uncertainty about balancing patient privacy with data needs for medical research and quality improvement, consistent with previous findings by [101].

The relationship between privacy protection and healthcare functionality emerged as a critical concern. While privacy measures like encryption and access controls are necessary, 93 survey participants indicated they could impede efficient workflows due to time pressure and workload constraints. However, healthcare professionals indicated willingness to adopt additional privacy measures if properly integrated into workflows and clearly communicated in terms of patient benefits.

Patient perspectives received insufficient attention in both research and practice. Our review included only 29 publications focused on patient perspectives and ethical considerations, despite their critical importance. Studies demonstrated that patients frequently lack adequate information about data processing [33], while survey participants reported challenges in providing transparent privacy information to patients. This communication gap potentially undermines patient trust and engagement in privacy protection.

## RQ1: Protection of Patient Data Privacy in Hospital eHealth Systems

The findings from the scoping review provide a comprehensive overview of how patient data privacy is addressed within hospital eHealth systems. Across 434 analyzed publications, most research concentrated on technical mechanisms such as blockchain, encryption, and artificial intelligence, reflecting a strong emphasis on technological innovation as the primary means of ensuring data protection. However, organizational, legal, and patient-centered approaches were significantly less represented. The dominance of technological research illustrates a prevailing view of privacy protection as a primarily technical challenge, emphasizing confidentiality and data integrity through cryptographic and access control methods. Yet, the limited exploration of procedural, ethical, and training-related aspects indicates that privacy management in hospitals remains fragmented.

In hospital environments, privacy protection is operationalized through a mix of technical safeguards and institutional policies. The reviewed studies identified role-based access controls, identity management systems, and secure data storage solutions as core mechanisms. Complementary organizational measures (e.g., staff training, privacy governance structures, and dynamic consent models) were described as essential but inconsistently applied across institutions. The literature further highlighted regional differences: while European publications frequently referenced the GDPR and Privacy-by-Design principles, studies from Asia and North America placed greater emphasis on scalability and interoperability. In summary, the evidence demonstrates that while hospitals increasingly adopt advanced privacy technologies, effective protection of patient data still depends on coherent integration with legal frameworks, staff awareness, and patient participation.

## RQ2: Discrepancies Between Theory and Practice

The survey results revealed a persistent gap between theoretical models of privacy protection and their practical implementation within hospital contexts. While theoretical frameworks advocate comprehensive, multilayered approaches combining technology, law, and ethics, practitioners reported difficulties translating these principles into routine operations. The majority of respondents associated privacy primarily with confidentiality and data security, demonstrating a narrower, operational understanding than conceptualized in academic literature. Reported implementation barriers included time pressure, unclear internal guidelines, insufficient training, and technical constraints of legacy systems. These findings expose an organizational bottleneck that hinders the realization of privacy principles despite regulatory mandates.

Furthermore, healthcare professionals expressed uncertainty about institutional responsibilities and accountability mechanisms in data protection, indicating incomplete internalization of legal and procedural standards such as those established under the GDPR. Although encryption and access controls were widely implemented, participants identified limited awareness and uneven enforcement across departments. This contrasts sharply with theoretical models emphasizing continuous education, organizational culture, and participatory governance. The discrepancy therefore lies not in the absence of technology, but in the lack of systemic alignment between technical measures, institutional routines, and professional behavior. Bridging this divide requires operational frameworks that translate abstract privacy principles into workflow-compatible, resource-sensitive practices across hospital settings.

## Bridging theory and practice - an integrated framework

Based on our integrated findings, we propose an exemplary framework with potential examples for enhancing privacy in eHealth systems that addresses the identified gaps between theoretical approaches and practical implementation:

**1. Enhanced training and awareness:** Implement regular, role-specific privacy training addressing technological, legal, and ethical aspects of data protection [31].

**Implementation Example**

- **High-resource:** Annual, role-specific eLearning modules with scenario-based simulations.
- **Low-resource:** Quarterly in-person briefings integrated into staff meetings.

**Responsible actors:** Data Protection Officer (DPO), department heads.

**Metrics:** Training completion rate ≥90%; post-training knowledge score improvement > 20%.

**2. Privacy-by-Design implementation:** Integrate privacy considerations from the inception of technologies and workflows, establishing clear privacy requirements for technology procurement.

**Implementation Example**

- **High-resource:** Inclusion of Privacy-by-Design checklists in IT procurement and vendor contracts.
- **Low-resource:** Template-based privacy impact assessments before system changes.

**Responsible actors:** IT management, procurement units, DPO.

**Metrics:** Share of new systems with documented privacy assessment; number of privacy-related incidents per quarter.

**3. Workflow-integrated measures:** Design privacy solutions that enhance rather than impede clinical processes, minimizing additional burdens on healthcare staff.

**Implementation Example**

- **High-resource:** Automated access control dashboards integrated into EHRs.
- **Low-resource:** Standardized paper/electronic logs for access justification.

**Responsible actors:** Clinical unit managers, IT administration.

**Metrics:** Audit compliance rate; average time to resolve access anomalies.

**4. Resource-appropriate approaches:** Develop scalable solutions adaptable to varying organizational capacities, with simplified implementation pathways for resource-constrained environments.

**Implementation Example**

- **High-resource:** Dedicated privacy budgets and staff positions.
- **Low-resource:** Regional privacy officer shared among institutions.

**Responsible actors:** Hospital administration, health authorities.

**Metrics:** Ratio of privacy budget to IT budget; number of privacy tasks completed per quarter.

**5. Patient-centered communication:** Create accessible information materials explaining privacy practices and engaging patients as active participants in data protection.

**Implementation Example**

- **High-resource:** Interactive patient portals displaying data-use logs.
- **Low-resource:** Printed one-page privacy summaries at admission.

**Responsible actors** Patient relations units, communications departments.

**Metrics:** Patient privacy-awareness survey scores; reduction in patient complaints on data handling.

**6. Regular evaluation mechanisms:** Implement standardized assessment procedures evaluating compliance, effectiveness, and staff acceptance of privacy measures [14].

**Implementation Example**

- **High-resource:** Annual privacy performance dashboard with benchmarking.
- **Low-resource:** Semi-annual audit checklist covering essential privacy measures.

**Responsible actors:** Compliance officers, quality management units.

**Metrics:** Number of corrective actions implemented; audit findings resolved within three months.

This integrated framework addresses both technical and human factors in privacy protection, potentially reducing the substantial gap between theoretical solutions and practical implementation identified in our research.

## Limitations

Several limitations should be considered when interpreting the findings of this study. First, the methodological constraints affected both components of our research. In the scoping review, the categorization of publications inevitably involved subjective judgment, which could influence the distribution of publications across thematic areas. Second, our search parameters introduced certain restrictions. Limiting the search to four databases, English-language publications, and studies published from 2021 onward may have excluded relevant insights and should be regarded as a methodological constraint that potentially limits the comprehensiveness of the review. Third, survey-related limitations affected the representativeness of our findings. The self-selected nature of participation likely introduced response bias, with individuals more concerned about privacy issues potentially overrepresented in our sample. Additionally, the non-probability sampling approach, relying on purposive and convenience recruitment within specific hospital networks, may have introduced sampling bias, limiting the generalizability of results to broader healthcare populations or other institutional contexts. Fourth, geographical limitations constrain the applicability of our results. The predominance of participants from Switzerland and Germany reflects privacy perspectives shaped by European regulatory frameworks, particularly the GDPR. This European focus, contrasting with the global distribution of publications in our scoping review, creates challenges in reconciling theoretical approaches with practical perspectives from similar regulatory contexts. Fifth, another limitation is that existing systematic and scoping reviews were excluded from the primary analysis. While these reviews were used to refine the search strategy and thematic framework, their exclusion as analytical sources may have limited direct engagement with higher-level syntheses of prior research. Consequently, some meta-level insights into overarching trends may not have been fully captured. Sixth, this scoping review did not systematically categorize or quantify study designs across included articles, precluding sensitivity analyses to assess whether specific designs influenced the identified themes. As scoping reviews aim to map the scope of evidence rather than examine design-specific effects, this represents a limitation that should be considered when interpreting the findings. Seventh, our study design did not include direct patient perspectives, relying instead on healthcare professionals’ perceptions of patient concerns. Finally, the scoping review and the analysis of the survey results were performed by only one expert reviewer. While a structured screening protocol and iterative calibration were applied, the absence of independent dual screening and formal agreement assessment may have introduced subjective bias. The use of an AI-based assistant (i.e., ChatGPT GPT-4o) does not substitute for independent human review and should be interpreted as a supportive measure rather than a validation mechanism. This limitation should be considered when interpreting the comprehensiveness and categorization of the findings.

Despite these limitations, the complementary approach combining literature analysis with healthcare professionals’ perspectives offers valuable insights into the current state of privacy protection in hospital eHealth systems and provides a foundation for future research addressing the identified gaps.

## Conclusion

This analysis examined privacy challenges and solutions in hospital eHealth systems through a dual approach combining a scoping review of 434 publications and a survey of 122 healthcare professionals. Our findings demonstrate that effective privacy protection requires integrating technological, organizational, legal, and patient-centered dimensions. The analysis revealed a significant gap between theoretical solutions and practical implementation challenges. Our investigation showed that organizational measures, legal frameworks, and patient perspectives are underrepresented in current research. Healthcare professionals emphasized the importance of designing privacy measures that accommodate clinical workflows rather than impeding them. The survey revealed that while practitioners recognize privacy’s importance, they reported a lack of awareness of privacy issues and knowledge of privacy measures, highlighting implementation challenges. Based on the findings, implementation examples for different resource settings to help translate privacy principles into actionable steps are proposed in the context of a practical framework. Future research should focus on developing integrated frameworks that balance theoretical rigor with practical implementation considerations, particularly addressing workflow integration and resource constraints. By bridging the gap between privacy theory and practice identified in this study, healthcare organizations can more effectively safeguard patient data while supporting digital health innovation.

## Supporting information

S1 Data Survey Questionnaire and Results. (XLSX)

S2 Data Survey Structure. (DOCX)

S3 Data Scoping Review Result. (XLSX)

S4 Data Scoping Review Results - Excluded Sources. (XLSX)

S1 Checklist PRISMA Checklist. (DOCX)

S2 Checklist Data Extraction Template. (DOCX)
