# Supplementary material for: Safeguarding patient privacy in eHealth systems: Bridging theory and practice through a scoping review and healthcare survey
Source: PLOS Digit Health. 2026 Mar 26;5(3):e0001325. doi: 10.1371/journal.pdig.0001325 (PMC13021168; doi:10.1371/journal.pdig.0001325)
Supplement: S2 Data — (DOCX) [file pdig.0001325.s002.docx]

1. Do you work in a hospital?

☐ Yes

☐ No

2. Which of the following best describes your profession?

☐ Profession in the medical field

☐ Profession in the medical-technical field

☐ Profession in the IT field

☐ Profession in the organizational/administrative field

☐ Other

3. To what extent do you see yourself as responsible for protecting patient data in your current professional role?

(Please rate on a scale from 1 = Not responsible at all to 5 = Very responsible)

4. How long have you been working in the hospital?

☐ Less than 1 year

☐ 1–5 years

☐ 6–10 years

☐ 11–20 years

☐ More than 20 years

5. In which country do you work?

☐ Germany

☐ Switzerland

☐ Austria

☐ Other

Understanding of Data Protection

6. What do you understand by data protection in the context of digital health applications? (Multiple answers possible)

☐ Confidentiality of patient data

☐ Protection against unauthorized access

☐ Ensuring data integrity

☐ Anonymization of health data

☐ Other

7. How important do you consider data protection in healthcare?

(Scale from 1 = Unimportant to 5 = Very important)

8. Why do you consider the protection of patient data important?

☐ Protection of sensitive personal data

☐ Trust between patients and healthcare personnel

☐ Compliance with legal regulations

☐ Preservation of human dignity

☐ Prevention of security risks

☐ Other

Measures to Protect Data

9. Which of the following measures are you aware of in your hospital? (Multiple answers possible)

☐ Encryption of patient data

☐ Two-factor authentication

☐ Regular data protection training

☐ Access controls

☐ Monitoring and logging of access

☐ Other

10. How do you rate the effectiveness of the current measures?

(Scale from 1 = Ineffective to 5 = Very effective)

11. What additional measures would you suggest to improve data protection?

Your answer: ______________________________________

Assessment and Experience

12. Have you ever experienced data protection breaches in the hospital?

☐ Yes

☐ No

13. How was the situation handled? (Multiple answers possible)

☐ Internal investigation

☐ Report to the data protection officer

☐ Report to the supervisor

☐ Technical adjustment or modification

☐ Staff training

☐ Not known to me

☐ Other

14. How would you assess the awareness and sensitivity of your colleagues regarding data protection?

(Scale from 1 = Very low to 5 = Very high)

15. How well informed do you feel about privacy measures and policies?

(Scale from 1 = Poorly informed to 5 = Very well informed)

16. How highly do you prioritize data protection compared to other tasks?

(Scale from 1 = Very low priority to 5 = Very high priority)

17. What challenges make data protection more difficult? (Multiple answers possible)

☐ Lack of training

☐ Technical limitations

☐ Time pressure and workload

☐ Unclear policies and processes

☐ Lack of support from management

☐ Communication problems within the team

☐ Other

18. How could your employer better support data protection?

Your answer: ______________________________________
