# Supplementary material for: Safeguarding patient privacy in eHealth systems: Bridging theory and practice through a scoping review and healthcare survey
Source: PLOS Digit Health. 2026 Mar 26;5(3):e0001325. doi: 10.1371/journal.pdig.0001325 (PMC13021168; doi:10.1371/journal.pdig.0001325)
Supplement: S2 Checklist — (DOCX) [file pdig.0001325.s006.docx]

**General Information**

- **Title**
- **Year of Publication**
- **Country**
- **Type of Study**
- **Categorization:**
  - Technical measures and innovations
  - Organizational measures and processes
  - Legal and regulatory aspects
  - Patient perspective and ethical considerations
- **What challenges are addressed with regard to privacy?**

**Technical Measures and Innovations**

- **Is a specific technology described?**
  - Blockchain
  - Encryption
  - AI/ML
  - IoT
  - Federated Learning
  - mHealth
  - Other
- **In which application area is the technology used?**

**Organizational Measures and Processes**

- Are challenges or barriers in the implementation of privacy measures addressed?
- Are there any indications of how these barriers can be overcome?
- Are internal privacy guidelines described in the publication?
- What organizational measures are described?

**Legal and Regulatory Aspects**

- **What is the legal focus of the publication?**
  - Legal framework
  - Compliance and implementation
  - International and regional differences
  - Privacy by design
  - Consent management
  - Sanctions and legal consequences
  - Innovation and legal challenges
  - Other
- **Which regulations are mentioned?**
- **Are challenges to compliance with national privacy laws addressed?**

**Patient Perspective and Ethical Considerations**

- **Which patient-oriented or ethical focus is addressed in the publication?**
  - Patient rights and autonomy
  - Transparency and trust
  - Consent management
  - Conflicts between privacy and research
  - Discrimination and fairness
  - Confidentiality and protection of sensitive data
  - Ethical guidelines and recommendations
  - Long-term impact on the doctor-patient relationship
  - Other
- **Which ethical or patient-oriented challenges are described?**
